# The Intolerance of Uncertainty Inventory: Validity and Comparison of Scoring Methods to Assess Individuals Screening Positive for Anxiety and Depression

**DOI:** 10.3389/fpsyg.2018.00388

**Published:** 2018-03-26

**Authors:** Marco Lauriola, Oriana Mosca, Cristina Trentini, Renato Foschi, Renata Tambelli, R. Nicholas Carleton

**Affiliations:** ^1^Department of Social and Developmental Psychology, Sapienza University of Rome, Rome, Italy; ^2^Department of Dynamic and Clinical Psychology, Sapienza University of Rome, Rome, Italy; ^3^Department of Psychology, University of Regina, Regina, SK, Canada

**Keywords:** intolerance of uncertainty, Intolerance of Uncertainty Inventory, confirmatory factor analysis, bifactor model, clinical validity, anxiety, depression, transdiagnostic

## Abstract

Intolerance of Uncertainty is a fundamental transdiagnostic personality construct hierarchically organized with a core general factor underlying diverse clinical manifestations. The current study evaluated the construct validity of the Intolerance of Uncertainty Inventory, a two-part scale separately assessing a unitary Intolerance of Uncertainty disposition to consider uncertainties to be unacceptable and threatening (Part A) and the consequences of such disposition, regarding experiential avoidance, chronic doubt, overestimation of threat, worrying, control of uncertain situations, and seeking reassurance (Part B). Community members (*N* = 1046; Mean age = 36.69 ± 12.31 years; 61% females) completed the Intolerance of Uncertainty Inventory with the Beck Depression Inventory-II and the State-Trait Anxiety Inventory. Part A demonstrated a robust unidimensional structure and an excellent convergent validity with Part B. A bifactor model was the best fitting model for Part B. Based on these results, we compared the hierarchical factor scores with summated ratings clinical proxy groups reporting anxiety and depression symptoms. Summated rating scores were associated with both depression and anxiety and proportionally increased with the co-occurrence of depressive and anxious symptoms. By contrast, hierarchical scores were useful to detect which facets mostly separated between for depression and anxiety groups. In sum, Part A was a reliable and valid transdiagnostic measure of Intolerance of Uncertainty. The Part B was arguably more useful for assessing clinical manifestations of Intolerance of Uncertainty for specific disorders, provided that hierarchical scores are used. Overall, our study suggest that clinical assessments might need to shift toward hierarchical factor scores.

## Introduction

Uncertainty can be a significant psychological and physiological stressor. Difficulties with uncertainty have been associated with ineffective coping, neuroticism, need for predictability, and cognitive reactions to ambiguity (e.g., rigid dichotomizing into fixed categories, seeking certainty, and resorting to “black-white solutions”) (Berenbaum et al., 2008; Rosen et al., 2014; Lauriola et al., 2015; McEvoy and Erceg-Hurn, 2015; Carleton, 2016b). Intolerance of Uncertainty (IU) is an “individual’s dispositional incapacity to endure the aversive response triggered by the perceived absence of salient, key, or sufficient information, and sustained by the associated perception of uncertainty” (Carleton, 2016b, p. 31). IU is a latent multidimensional construct, reflecting fear of the unknown (Hong and Cheung, 2015; Carleton, 2016a). Substantial evidence indicates IU is a transdiagnostic factor for diverse psychopathology (Carleton et al., 2012; Mahoney and McEvoy, 2012; Einstein, 2014; Carleton, 2016a), with higher scores in clinical populations across disorders (Holaway et al., 2006; Gentes and Ruscio, 2011; Sternheim et al., 2011) and proportionate increases with comorbidity (Holaway et al., 2006; Yook et al., 2010; McEvoy and Mahoney, 2011).

The *Intolerance* of *Uncertainty Inventory* (IUI; Gosselin et al., 2008; Carleton et al., 2010) is a new comprehensive IU scale. Different from other IU scales, the IUI is comprised of two sets of items that can be administered together or separately. The first set (IUI-A; General Unacceptability of Uncertainty) was developed to assesses core beliefs about IU as currently defined (Carleton, 2016b). Accordingly, IUI-A items were devised as a coherent set of statements tapping into the tendency for the person to consider uncertainties in life to be unacceptable and threatening (e.g., “Not knowing what will happen in advance is often unacceptable for me”). Importantly, these beliefs were added later to the theoretical definition of the IU construct and were not specifically addressed in the classic IUS scales (Carleton, 2016b). The second set of items (IUI-B; Negative Manifestations of Uncertainty) was devised to cover six specific consequences of IU, which are common to observe in clinical patients, across different affective disorders.

*Worrying* may be the most common IU consequence included in the IUI-B (e.g., “Uncertain situations worry me”). Patients with GAD report ongoing worry helps them prepare to cope with unpredictable negative events (Dugas et al., 1998; Newman et al., 2013). High IU potentiates *overestimation of threat* operatively defined in the IUI-B as the tendency to exaggerate the probability that a negative event will occur (e.g., “In an uncertain situation, I tend to exaggerate the chances that things may go badly”). Chronic IU is associated with doubt, a hallmark feature of Obsessive Compulsive Disorder (OCD) (Nikodijevic et al., 2015; Samuels et al., 2017); accordingly, the IUI-B includes *doubting* items to assess absent confidence in thoughts, judgments, actions, and feelings (e.g., “When I am uncertain, I tend to doubt my capabilities”). Patients with GAD and OCD report desires to control uncertainty and therein defuse short-term anxiety and discomfort (e.g., compulsions in OCD, safety behaviors in GAD); as such, the IUI-B includes items assessing *need for control* (e.g., “I prefer to control everything in order to decrease uncertainties”). When worrying and control are insufficient, high IU may cause *reassurance seeking* from others or authoritative sources, as measured by the IUI (e.g., “When I am uncertain, I need to be reassured by others”); paradoxically, seeking reassurance can maintain anxiety symptoms over time (e.g., Kobori and Salkovskis, 2013). Finally, patients with high IU may engage in *avoidance* to cope, which typically produces only short-term reductions in anxiety (Sexton and Dugas, 2008; Mahoney et al., 2016). The IUI-B avoidance items assess attempts to escape uncertainty (e.g., “I tend not to engage in activities involving some uncertainty”).

Sound methods for separately assessing IU core beliefs and the clinical consequences of IU are useful for ascribing the positive consequences of clinical interventions to changes in beliefs as well as identifying specific targets to prioritize in clinical practice. Nevertheless, the IUI has not been extensively used in clinical research, nor has the IUI factorial structure been cross validated beyond north-American borders. Existing evidence generally supported a unidimensional structure for the IUI-A, and a six-factor structure for the IUI-B reflecting the aforementioned consequences of IU (Gosselin et al., 2008; Carleton et al., 2010). However, these findings were not unequivocal. Although a unidimensional structure was acceptable, the first study (Gosselin et al., 2008) concluded that a three-factor structure [(I) intolerance of uncertainty and uncertain situations; (II) intolerance of the unexpected; (III) difficulty waiting in an uncertain situation] best represented the IUI-A. Regarding IUI-B, the same study showed that the hypothesized six-factor structure [(I) avoidance; (II) doubt; (III) overestimation; (IV) worry; (V) control; and (VI) reassurance] was an excellent fit to the data. The second study (Gosselin et al., 2008) showed that the fit indices for the IUI-A were unacceptable both for the unidimensional factor model and for the multifactor model. The unitary factor model was trimmed based on the modification indices, and the atheoretical removal of items #2, #9, and #13 improved the model fit. The same study also showed that the fit indices supported the six-factor model for the IUI-B, but did not meet the acceptable standards (Carleton et al., 2010). As a whole, these results underscore the need for a cross-validation study of IUI factors on independent samples in different languages as well as for some psychometric refinements of the IUI scoring system.

The current study was primarily designed to assess the factor structure of the IUI-A and IUI-B using the models proposed in the extant literature as well as testing new hierarchical models for the IUI-B. For the IUI-A, we started with testing a unidimensional model to replicate the overall tendency for IU core beliefs items to reflect a unitary core dimension, and then followed up this analysis to assess the impact of removing critical items, as proposed in the literature (Carleton et al., 2010). For the IUI-B, previous research did not find recognizable solutions within modification indices. Nevertheless, hierarchical factor models were not fitted to the IUI-B item set, although this class of models is more appropriate to represent multifaceted personality constructs. First, we proposed a second-order factor model in which a general IU factor influences item responses through the six IUI-B first-order factors. Theoretically, the second-order model assumes that general IU (i.e., “a latent fear of the unknown”; Carleton, 2016a) will not directly influence the behavioral manifestations of IU; instead, general IU effects are expected to be mediated by more proximal first-order factors (i.e., avoidance, doubting, overestimation, worrying, control, and reassurance). Second, we assessed a bifactor model in which a general IU factor does directly influence IUI-B items above and beyond the more proximal more proximal first-order factors.

Multifaceted scales should also be assessed for the relative utility of the total and subscale scores in clinical assessments. General and specific variance proportions are variably entangled, complicating the extent to which clinical groups may differ on a general trait (e.g., ‘a latent fear of the unknown’ for IU multidimensional assessment scales) or on a specific manifestation of that trait (e.g., ‘overestimation of threat,’ ‘need for control’). Hierarchical factor models offer clinical researchers an opportunity to derive factor scores that parse general and specific variance (Reise et al., 2010; Chen et al., 2012). The current study compares aggregated IUI scores and hierarchical factor scores for assessing individuals screening positive for anxiety and depressive disorders, which are highly comorbid and critically associated with IU (Miranda et al., 2008; Carleton et al., 2012; Mahoney and McEvoy, 2012; Carleton, 2016a). Comparing scoring methods may provide insights for the co-occurrence of depressive and anxious symptoms. In clinical groups, elevated subscale scores may be due to higher general distress rather than IU-specific mechanisms. Profile elevations across subscales may be due to entanglement with general IU factor variance. Accordingly, we hypothesized that IUI summated ratings might produce divergent response patterns between participants who were above the clinical cut-offs for anxiety and depression and those who were not (Holaway et al., 2006; Gentes and Ruscio, 2011; Sternheim et al., 2011), and aggregated ratings proportionally increase with the co-occurrence of depressive and anxious symptoms (Holaway et al., 2006; Yook et al., 2010; McEvoy and Mahoney, 2011). By contrast, we expect some divergent response patterns between the two groups using hierarchical factor scores. For example, some scores (e.g., worry, doubting) might best characterize individuals screening positive for anxiety disorders, while other scores (e.g., overestimation of threat) might best characterize those individuals screening positive for depression.

## Materials and Methods

### Participants and Procedures

The sample was based on convenience rather than randomly drawn from a target population; nevertheless, approximate quotas were set for age, gender and education to ensure heterogeneous sampling. Participants included 1046 community members (414 men, 627 women, 5 undisclosed gender) who completed a series of self-report measures as part of a larger study approved by the local ethical review board for psychological research. Participant ages ranged from 20 to 76 years (*M* = 36.69; *SD* = 12.31). Completed education levels were distributed as follows: senior high school (*N* = 454; 43.5%), junior high school (*N* = 464; 44.5%), and elementary school (*N* = 125; 12.0%). Eighty-nine undergraduate psychology students attending an advanced clinical assessment class were asked to recruit research participants among their acquaintances and to serve as interviewers. The third author of this paper trained all the students for standardization of questionnaire administration in small group sessions. Before data entry, the third author debriefed the students and verified the accuracy of the collected data. No special problems were encountered but sporadic missing data. Other psychology students or close family members of the recruiter were excluded from the study. The questionnaires were administered at home in a quiet and comfortable room. Each interviewer acquainted potential participants with the study goals, the voluntary nature of participation, the right to withdraw from the study at any moment, and that responses would be kept anonymous once submitted. Verbal consent was obtained from each participant before data collection. The data were collected over a 3-week period, and each interviewer collected a variable number of cases (ranging from 6 to 31) on a voluntary base.

### Measures

#### Intolerance of Uncertainty Inventory

Participants completed the 45-item version of the IUI (Gosselin et al., 2008), containing 15 items for IUI-A and 30 items for IUI-B. The IUI items were translated into Italian by the first and the second author for use in the current study. Then, a bilingual professional translator, without reference to the original text, back-translated the IUI into English to verify linguistic equivalence. Minor discrepancies between translations were resolved through discussion. Following Gosselin et al. (2008), the items were administered using a 5-point Likert scale ranging from 1 (‘not at all characteristic of me’) to 5 (‘entirely characteristic of me’). The Italian version of the IUI items is reported in the Supplementary Table 1. Scoring key and descriptive statistics are reported in the Supplementary Table 2.

#### Beck Depression Inventory II

The Beck Depression Inventory II (BDI-II; Beck et al., 1996; Italian version, Ghisi et al., 2006) is a 21-item multiple-choice self-report scale. The BDI-II was designed to assess affective, somatic, or cognitive symptoms of depression. Respondents task was to rate the severity of each symptom using a 4-point Likert scale ranging from 0 to 3 (higher numbers indicated greater severity). The total score (α = 0.89, in the present study) is a valid measure of the severity of depression. Total scores of 0–13 indicates ‘minimal or no depression.’ Total scores ranging from 14–19, 20–28, and 29–63 are used to classify participants as reporting ‘mild,’ ‘moderate,’ and ‘severe’ depression levels, respectively.

#### State-Trait Anxiety Inventory

State-Trait Anxiety Inventory (STAI-Y; Spielberger, 1983; Italian version, Pedrabissi and Santinello, 1989) is a 40-item self-report measure designed to assess transitory and chronic anxiety symptoms. The 20-item trait subscale was used in the present study. The total score (α = 0.93, in the present study) is considered a valid measure of trait neuroticism, that is the tendency to chronically experience a wide range of negative affect states (e.g., fear, worry, autonomic nervous system somatic symptoms). The recommended clinical cut-off score for the A-trait total score is > 46 (Fisher and Durham, 1999), which is how the A-trait scale was used to separate high trait anxious individuals from the rest of the sample.

### Data Analysis

#### Missing Values

Sporadic missing values were imputed using a random Hot Deck (Andridge and Little, 2010). Accordingly, we replaced each missing value in an item with an individual response from a similar case picked at random from those in the dataset (i.e., same age, gender, and education).

#### CFA Models

Structural equation modeling (EQS 6.2; Bentler, 2004) was used to assess the factorial structure of the IUI. Separate analyses were carried out for IUI-A and IUI-B. The data deviated from the assumptions of multivariate normality (i.e., Mardia’s normalized coefficient = 46.18 and 113.85, respectively, for the IUI-A and IUI-B datasets); accordingly, the Maximum Likelihood Robust method (MLR) was used to adjust model parameters and fit. In line with previous research (Gosselin et al., 2008; Carleton et al., 2010), we tested single-factor, two-factor, and three-factor models for the IUI-A, as well as three-factor and six-factor models for the IUI-B. Recent evidence and theory have suggested that IU was best modeled as hierarchical multifaceted construct (Hale et al., 2016; Lauriola et al., 2016); accordingly, we also tested second-order and bifactor models for the IUI-B, in which a general IU factor loaded all items, while six independent group factors loaded on avoidance, doubt, overestimation, worry, control and reassurance items, respectively.

#### Assessment of Model Fit

Model fit was assessed using the following indices: Satorra–Bentler scaled χ^2^ (SBχ^2^), robust versions of Comparative Fit Index (CFI), Bentler–Bonnett Non-Normed Fit Index (NNFI), Root Mean Square Error of Approximation (RMSEA) and Standardized Root Mean Square Residual (SRMR). According to Hu and Bentler (1999), cutoff values close to 0.95 for NNFI and CFI, close to 0.06 for RMSEA, and close to 0.08 for SRMR are needed to conclude that there is a relatively good fit between the factor model and the data.

#### Model Comparisons

Nested factor models are models that can be derived one from the other by estimating fewer parameters. For example, a single factor model is nested in a two-factor model that insists on the same observed variables, so that the former can be obtained from the latter by constraining the correlation between the two latent variables to 1.00 (i.e., the single factor model has one parameter less than the two-factor model). Nested models can be compared statistically with a chi-square difference test to assess whether the model restrictions significantly impacted fit. For comparisons that are not statistically significant the more restrictive model is preferred. Conversely, the less restrictive model is preferred for statistically significant comparisons. In using MLR for the current study the chi-square difference test was corrected per Satorra and Bentler (2001) formula.

Non-nested models that insist on different subsets of observed variables can also be compared (e.g., dropping items with poor fit from subsequent CFA analyses) using ‘information criteria’ indices that adjust the ML fit functions based on the number of parameters. The Consistent Akaike Information Criterion (CAIC; Bozdogan, 1987) is considered the preferred index for such analyses but has no intuitive value for interpretation and no recommended cut-off scores. Lower CAICs are associated with a higher likelihood that the tested model approximates the ‘true’ model, thereby having greater chances to be replicated in subsequent cross-validation studies.

#### Reliability Analyses

For standard factor models, like the IUI-A single-factor model or the IUI-B six-factor model, the coefficient omega (ω) was used to assess the proportion of reliable variance in the set of observed variables that was accounted for by each latent variable in the model. For hierarchical models, in which each observed variable reflects both common and unique amounts of reliable variance, measurement equations were used to assess the relative contribution of each amount. The reliability coefficient omega was computed for the total score that, in second-order or bifactor models, reflects the proportion reliable variance that was accounted for by both the general and the group factors. The omega hierarchical coefficient (ω_h_) was used to assess the proportion of variance accounted for by the general factor only in the total score. Where ω_h_ is appreciably different from ω, the reliable variance in the total score reflects the general factor as well as the group factors (Reise, 2012). The omega (ω) and omega scale (ω_s_) coefficients can also be compared to assess the viability of subscale scores with group-factor items (Reise, 2012). Whereas ω reflects a mixture of general and unique variance for any specific subscale, ω_s_ is a measure of subscale reliability after the general factor variance has been partialed out. If ω_s_ is as large as ω, then the subscale score reflected mostly the group factor reliable variance. Most commonly ω_s_ tend to be smaller than ω as the common variance is greater.

#### Validity Analyses

Participant responses on the clinical scales for anxiety and depression were screened into positive and negative groups. The screening was based on internationally established cut-offs (i.e., BDI-II and STAI scores greater than 13 and 46, respectively). The delineation allowed for comparisons of IUI responses patterns for the IUI-A and IUI-B between groups, either using summated ratings or bifactor model scores. Hierarchical factor scores were computed using the Anderson-Rubin method, which ensures the orthogonality of the estimated factors and produces scores that have a mean of 0, and a standard deviation of 1 (Distefano et al., 2009; Revelle, 2017).

## Results

### CFA Results of IUI-A

We first examined the fit indices for factor models proposed elsewhere for the IUI-A (Gosselin et al., 2008). As detailed in **Table 1**, the single factor model was statistically significant and the fit indices were inconsistent with the recommended standards (Hu and Bentler, 1999); nevertheless, all items loaded onto the latent factor significantly and the composite reliability coefficient for the total score was high (ω = 0.92). Similarly, the two-factor model with “intolerance of uncertainty and of uncertain situations” (i.e., items 1, 2, 3, 4, 5, 8, 9, 11, 15) and “intolerance of the unexpected and difficulty waiting in an uncertain situation” (i.e., items 6, 7, 10, 12, 13, 14) (Gosselin et al., 2008, p. 1434) were inconsistent with the recommended standards (**Table 1**); moreover, the two latent variables were too highly inter-correlated (*ϕ* = 0.98) to support meaningful distinctions. The three-factor model with “intolerance of uncertainty and of uncertain situations” (i.e., items 1, 2, 3, 4, 5, 8, 9, 11, 15), “intolerance of the unexpected” (i.e., items 7, 14), and “difficulty waiting in an uncertain situation” (i.e., items 6, 10, 12, 13)” (Gosselin et al., 2008, p. 1434) were also inconsistent with the recommended standards (**Table 1**). The latent variables for the three-factor model were again highly inter-correlated (*ϕ*-s > 0.91), suggesting against that solution for the IUI-A.

**Table 1:** Fit indices for the confirmatory factor analytic models of IUI-A and IUI-B.

Model	*SBχ^2^* (*df*)	*NNFI*	*CFI*	*SRMR*	*RMSEA* (95% CI)
IUI-A, Single Factor	947.36^∗∗^ (92)	0.874	0.890	0.067	0.094 (0.089–0.100)
IUI-A, Two Factor	922.28^∗∗^ (89)	0.873	0.893	0.063	0.095 (0.089–0.100)
IUI-A, Three Factor	865.28^∗∗^ (87)	0.879	0.900	0.062	0.093 (0.087–0.098)
IUI-A, Two Factor, 5-item Latent Variable	858.45^∗∗^ (89)	0.883	0.901	0.068	0.091 (0.085–0.096)
IUI-A, Single Factor, 10-items	338.14^∗∗^ (35)	0.932	0.947	0.064	0.091 (0.082–0.100)
IUI-B, Six Factor	1405.24^∗∗^ (390)	0.932	0.939	0.043	0.051 (0.043–0.053)
IUI-B, Three Factor	948.18^∗∗^ (167)	0.921	0.931	0.050	0.068 (0.063–0.072)
IUI-B, Second Order Model	1520.03^∗∗^ (399)	0.927	0.933	0.069	0.053 (0.050–0.055)
IUI-B, Bifactor Model	1331.22^∗∗^ (375)	0.934	0.943	0.041	0.050 (0.047–0.053)

*NNFI, robust version of non-normed fit index; CFI, robust version of comparative fit index; RMSEA, robust version of root mean square error of approximation; SRMR, standardized root mean square residual; IUI-A, intolerance of uncertainty index part A; IUI-B, intolerance of uncertainty index part B; ^∗∗^*p* < 0.001.*

For the standardized factor loadings for the single factor IUI-A model, ten items had coefficients greater than 0.60 and five items (i.e., 1, 2, 3, 6, 15) had relatively lower loadings (i.e.,.50,.59,.59,.42, respectively). We tested a new model with a second latent variable using these five items. The results were statistically significant and significantly improved Model’s fit relative to the single factor model, Δ*SBχ^2^* = 186.19 (*df* = 1; *p* < 0.001) and the two-factor model proposed by Gosselin et al. (2008, p. 1434), Δ*SBχ^2^* = 63.83, (Δ*df* = 0); however, the fit indices still were inconsistent with the recommended standards (**Table 1**). The two latent variables were again very highly inter-correlated (ϕ = 0.83).

Overall, the IUI-A results supported a unitary factor structure consistent with previous research, but also advised to optimize the scale. The inspection of the standardized factor loading matrix suggested that one might remove the five items with the lower commonality (i.e., *h*^2^ < 0.36). The revised IUI-A single factor model after item removal was consistent with most of the recommended standards for all indices (**Table 1**). All items loaded significantly on the latent factor (all *λs* > 0.60) and the reliability coefficient omega for the total score with ten items was about as large as that assessed in the previous analysis (*ω* = 0.91). Accordingly, we used the ten-item IUI-A factor score in subsequent validity analyses (*M* = 27.78; *SD* = 9.38, in the present study).

### CFA Results of IUI-B

The IUI-B was designed to have a six-factor structure reflecting clinical manifestations of core IU beliefs, like ‘avoidance’ (i.e., Items 1, 8, 12, 22, 26), ‘doubt’ (i.e., Items 2, 7, 13, 21, 30), ‘overestimation’ (i.e., Items 3, 14, 19, 23, 29), ‘worry’ (i.e., Items 6, 15, 17, 20, 28), ‘control’ (i.e., Items 4, 10, 18, 24, 27), and ‘reassurance’ (i.e., Items 5, 9, 11, 16, 25). Accordingly, we started by testing that six-factor model with correlated latent variables. The resulting fit indices were consistent with the recommended standards for all indices (**Table 1**), and CAIC was -376.05; however, an alternative three-factor model has been proposed (Carleton et al., 2010), with the original ‘control’ and ‘overestimation’ factors plus a ‘manifestations of anxious thought’ broad factor subsuming ten items selected from the original doubt, reassurance, and worry factors (i.e., Items 2, 5, 6, 7, 9, 11, 13, 17, 21, and 30). The three-factor model also produced fit indices consistent with the recommended standards for all indices (**Table 1**), but the CAIC was -1684.97. Since smaller CAIC values indicate better fit, the three-factor model based on lesser items was preferred.

The six-factor model inter-factor correlations were high with *ϕ*-s ranging from 0.68 to 0.83, except for ‘doubt’ with ‘control’ factors (*ϕ* = 0.52). The IUI-B was designed as a multidimensional clinical tool and the current results support notions that IU clinical manifestations represent lower order facets of a multifaceted hierarchical model. We tested a second-order factor model in which a General IUI-B factor was posited to affect the various clinical manifestations or consequences of IU through the six first-order factors. The second-order factor model produced fit indices consistent with the recommended standards for all indices (**Table 1**); however, the model fitted significantly worse than the six-factor model, Δ*SBχ^2^* = 120.56 (*df* = 9; *p* < 0.001). A less constrained bifactor model, in which a common IUI-B factor was posited to affect the clinical manifestations or consequences of IU directly and independently from the six group factors, produced fit indices consistent with the recommended standards for all indices (**Table 1**). This model fitted significantly better than the six-factor model, Δ*SBχ^2^* = 75.38 (*df* = 15; *p* < 0.001), and appeared to be the most accurate IUI-B factorial structure representation for the current data.

The IUI-B can be scored by deriving a single total score for the general factor or six subscale scores for each of the group factors. Based on standardized factor loadings (**Table 2**), the reliability analyses described by Reise (2012) for bifactor model scores were used to assess the viability of total and sub-scale scores for IUI-B. First, we assessed the proportion of reliable variance in the total score accounted for by the general factor (*ω_h_* = 0.70) and compared that to total proportion of reliable variance (*ω* = 0.96). The general factor accounted for about 70% of the total score reliable variance, whereas the total score reliability was lower for the portion of reliable variance accounted for by group factors (i.e., ∼26%). We then compared the standard omega assessed for each group-factor items (*ω*) and the omega scale hierarchical (*ω_s_*) to assess the unique information conveyed by the IUI-B subscales. The ω_s_ provided a measure of reliability after partialing out the general factor variance was from the sub-scale scores. The standard omega coefficients for the six subscales were all fairly high for five-item scales (i.e., *ω =* 0.83 for avoidance, doubt, overestimation, worry, control; *ω* = 0.82 for reassurance). In contrast, ω_s_ coefficients fell – often substantially – for worry (*ω_s_ =* 0.11), doubt (*ω_s_* = 0.16), overestimation (*ω_s_* = 0.20), avoidance (*ω_s_* = 0.25), reassurance (*ω_s_* = 0.27), and control (*ω_s_* = 0.40). Overall, the IUI-B subscale scores reliably measured common variance in IU, but also maintained some specific amount of information relative to using the IUI-B total score, particularly for avoidance, reassurance, and control.

**Table 2:** Standardized factor loadings for the bifactor confirmatory factor analysis model of IUI-B.

Item	F1 Avoidance	F2 Doubting	F3 Overestimation	F4 Worrying	F5 Control	F6 Reassurance	F7 General
IUI-B 12	0.50	–	–	–	–	–	0.60
IUI-B 22	0.37	–	–	–	–	–	0.57
IUI-B 26	0.34	–	–	–	–	–	0.49
IUI-B 1	0.32	–	–	–	–	–	0.46
IUI-B 8	0.31	–	–	–	–	–	0.63
IUI-B 21	–	0.47	–	–	–	–	0.66
IUI-B 7	–	0.39	–	–	–	–	0.68
IUI-B 13	–	0.29	–	–	–	–	0.63
IUI-B 2	–	0.27	–	–	–	–	0.64
IUI-B 30	–	0.21	–	–	–	–	0.71
IUI-B 29	–	–	0.44	–	–	–	0.76
IUI-B 14	–	–	0.41	–	–	–	0.71
IUI-B 19	–	–	0.41	–	–	–	0.77
IUI-B 3	–	–	0.34	–	–	–	0.71
IUI-B 23	–	–	0.46	–	–	–	0.75
IUI-B 28	–	–	–	0.45	–	–	0.65
IUI-B 6	–	–	–	0.39	–	–	0.69
IUI-B 17	–	–	–	0.23	–	–	0.78
IUI-B 15	–	–	–	0.21	–	–	0.76
IUI-B 20	–	–	–	0.16	–	–	0.79
IUI-B 27	–	–	–	–	0.60	–	0.58
IUI-B 10	–	–	–	–	0.58	–	0.58
IUI-B 18	–	–	–	–	0.56	–	0.65
IUI-B 4	–	–	–	–	0.46	–	0.43
IUI-B 24	–	–	–	–	0.43	–	0.48
IUI-B 9	–	–	–	–	–	0.65	0.56
IUI-B 5	–	–	–	–	–	0.56	0.48
IUI-B 11	–	–	–	–	–	0.40	0.55
IUI-B 25	–	–	–	–	–	0.20	0.66
IUI-B 16	–	–	–	–	–	0.19	0.65

### Comparison of Summated Ratings and Hierarchical Factor Sores

**FIGURE 1:**
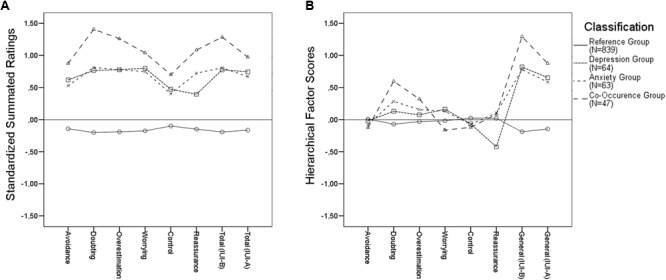
Intolerance of uncertainty profile for research participants classified according to BDI and STAI cut-off scores.

Using the established cut-offs for anxiety and depression scales, we identified *N* = 112 (10.7%) and *N* = 114 (10.9%) participants screening positive for chronic anxiety (STAI A-trait > 46) and moderate depression (BDI-II > 20), respectively. The STAI A-trait and BDI-II classifications were positively correlated (Spearman’s *Rho* = 0.36; *p* < 0.001). Accordingly, we reclassified the research participants into three clinical proxy groups: 63 cases (6.1% of the sample) scoring above the cut-off for chronic anxiety on the STAI A-trait, only; 66 cases (6.3%) scoring above the cut-off for moderate depression on the BDI-II, only; 48 cases (4.6%) scoring above the cut-off on both the STAI A-trait and the BDI-II. A reference group of 868 cases (83%) participants who scored below the clinical cut-off for both anxiety and depression, and were also identified for comparisons in data analyses. For simplicity, hereafter we refer to these groups as “anxiety,” “depression,” “co-occurrence,” and “reference” group, respectively. This classification was used as a between-subjects factor in two multivariate analyses of variance under the hypotheses that greater IU is associated with greater co-occurrence of depressive and anxious symptoms, and that depression and/or anxiety is associated only with specific clinical manifestations of IU. The first analysis compared the groups on the IUI summated ratings (**Figure 1A**). The second analysis compared the groups on the IUI-A standard factor score and on the IUI-B hierarchical factor scores estimated from the best fitting CFA models for each part of the inventory (**Figure 1B**). Divergent results between the analysis might reveal the extent to which group differences could be biased, and potentially misleading, when summated ratings are used to make inferences at the facet level for multifaceted hierarchical constructs.

The IUI-A and the IUI-B total scores were highly correlated both using summated ratings and factor scores (*r-s* = 0.76 and 0.74, respectively). Using summated ratings, the IUI-B total score were highly correlated with IUI-B subscale scores (*r-s* range 0.75–0.86); the coefficients were somewhat lower for IUI-A with IUI-B subscale scores (*r-s* range 0.75–0.86). Using factor sores, the IUI-B general factor was uncorrelated with IUI-B factor scores; specifically, the coefficients were significant only for the IUI general factor with worry (*r* = 0.20) and need for control (*r* = 0.07). This correlation analysis indicated that, using hierarchical factor scores, respondents can have IUI-B scores that parse specific and common sources of variance in ratings.

The analysis of summated ratings (**Figure 1A**) indicated a significant multivariate effect of the classification variable (Roy’s root = 0.298; *F* = 42.82; *df-*s = 8,1006; *p* < 0.001; $\eta_{p}^{2}$ = 0.23). Follow-up contrast analyses indicated that, when combined, the three clinical proxy groups were significantly higher than the reference group on all IUI-B subscales, as well as on the IUI-B and the IUI-A total scores (*Ψ*_avoidance_ = 2.45; *Ψ*_doubting_ = 3.57; *Ψ*_overestimation_ = 3.39; *Ψ*_worrying_ = 3.11; *Ψ*_control_ = 1.86; *Ψ*_reassurance_ = 2.64; *Ψ*_IUI-Btotal_ = 3.47; *Ψ*_IUI-Atotal_ = 2.89; all *p*-s < 0.001). The anxiety and depression groups were significantly lower than the co-occurrence group on some IUI-B subscales, and on both the IUI-B and IUI-A total score (*Ψ*_doubting_ = -1.26; *Ψ*_overestimation_ = -0.96; *Ψ*_reassurance_ = -1.06; *Ψ*_IUI-Btotal_ = -0.99; all *p*-s < 0.001); however, the anxiety and depression groups were not significantly different on any of the summated ratings scores.

The analysis of hierarchical scores (**Figure 1B**) also indicated a significant multivariate effect of the classification variable (Roy’s root = 0.293; *F* = 36.43; *df*s = 8,1006; *p* < 0.001; $\eta_{p}^{2}$ = 0.22). As in the analysis of summated ratings, the follow-up contrasts revealed that the three clinical proxy groups combined were significantly higher than the reference group on both the IUI-B and IUI-A general factor scores (*Ψ*_IUI-Bgeneral_ = 3.45; *Ψ*_IUI-Ageneral_ = 2.55; both *p*-s < 0.001); however, only some of the IUI-B factor scores yielded significant differences between clinical proxy groups combined and the reference group (*Ψ*_doubting_ = 1.22, *p* < 0.001; *Ψ*_overestimation_ = 0.64, *p* < 0.05). Regarding doubting scores, a follow up analysis indicated that the co-occurrence group was significantly higher than the depression and anxiety groups combined (*Ψ*_doubting_ = 0.78, *p* < 0.05), while these latter groups did not differ significantly. Instead, no combination of clinical proxy groups yielded statistically significant comparisons on overestimation of threat factor scores.

The anxiety and depression groups were also significantly lower than the co-occurrence group on the IUI-B general factor (*Ψ*_IUI-Bgeneral_ = -0.99; *p* < 0.01), and marginally on the IUI-A general factor (*Ψ*_IUI-Ageneral_ = 0.51; *p* = 0.07). The two clinical proxy groups of participants scoring above the cut-off either on anxiety or depression were statistically different on the reassurance group factor (*Ψ*_reassurance_ = 0.54; *p* < 0.01), but not on the doubting and overestimation scores. In particular, as detailed in **Figure 1B**, participants in the depression group were less apt than other clinical proxy groups to seek reassurance from other people or presumed authoritative sources in order to cope with feared unknowns, a result that would be overlooked using summated ratings instead of hierarchical scores.

## Discussion

The current study evaluated the validity of the IUI, a two-part scale separately assessing IU core beliefs (IUI-A) and the clinical consequences of these beliefs in diverse clinical disorders (IUI-B). The IUI-A was best explained by a unidimensional structure. Alternative multiple factor models proposed in the extant literature for French and English versions of the scale were not supported. Indeed, the hypothesis that items poorly loading on the single latent variable could give rise to a theoretically meaningful second latent factor was rejected due to the large empirical overlapping of the two latent variables in the models tested. Despite a unidimensional structure, however, the IUI-A produced the most robust fit indices for a single factor model in which the latent variable used only a 10-item subset of the original 15 items. Previous research also showed that an atheoretical removal of three items from the English language version improved the fit of a unitary solution for the IUI-A (Carleton et al., 2010). Nevertheless, the subset of items used in the present study was different from that used in previous studies. Previous research with Italian and English speaking participants has pointed out some caveats related to the use of IU scales across countries (Bottesi et al., 2015, 2016). Because the IUI-A factor structure was problematic in two different languages (i.e., French and English), as well as in the present study, while the IUI-B seemed more robust to cultural and translational issues, we believe that translation bias was not a significant problem in this study. We speculate that people with different cultural background may differ in how the cultures engage with uncertainty at the level of IU core beliefs (e.g., appraisal and acceptance of uncertain situations, discomfort with the unexpected, or difficulty waiting in an uncertain situation). By contrast, the structure of the clinical consequences of IU was approximately the same in French, English, and Italian studies, showing that reactions to uncertainty were comparable across cultures. The present findings add to the extant literature (Gosselin et al., 2008; Carleton et al., 2010) in that they reinforce the need for refining the assessment of IU core beliefs for the use of the IUI-A in cross-cultural research. The unitary factor structure of the IUI-A was supported overall, but the impact of removing items from the original set remains to be reassessed. In the present study, we proposed a 10-item version that calls for a cross-validation across languages (e.g., French and English) and cultures (e.g., North American and European countries). It is noteworthy, however, that the IUI-A total score with ten items was highly reliable and had fair criterion validity with BDI-II and STAI classifications as well as high convergent validity with the IUI-B.

Regarding the IUI-B, the intended six-factor structure produced robust fit indices in all countries and languages with avoidance, doubting, overestimation, worrying, seeking reassurance, and need for control factors (Gosselin et al., 2008; Carleton et al., 2010). Moreover, the factor analytic results supported the view that IU was best modeled as hierarchical multifaceted construct (Hale et al., 2016; Lauriola et al., 2016). A bifactor model with one general factor common to all the items, as well as the six factors common to specific groups of items, was evidenced as producing superior model fit indices relative to the standard six-factor model. In other words, the general factor captured the variance common to all items describing diverse clinical manifestations of IU in GAD, Depression and OCD patients, but each specific manifestation was also affected by a unique source of variance associated with specific groups of items. This result implies that the general IU factor may be contributing to a transdiagnostic range of disorders whereas the group factors may be contributing to specific disorders, or patients (Carleton et al., 2012; Mahoney and McEvoy, 2012; Einstein, 2014; Carleton, 2016b).

According to the view that IU is higher in clinical groups than in control groups across several disorders (Holaway et al., 2006; Gentes and Ruscio, 2011; Sternheim et al., 2011; Carleton et al., 2012), our study showed that both the IUI-A and IUI-B summated rating scores discriminated between clinical proxy groups and a reference group. The IUI-A and IUI-B summated rating scores were both higher among participants scoring above the cut-off on the two proxy measures of anxiety and depression, relative to those scoring above the cut-off on only one of the proxy measures; as such, the results were consistent with the view that IU proportionally increases with co-occurrence of depressive and anxious symptoms (Holaway et al., 2006; Yook et al., 2010; McEvoy and Mahoney, 2011). The overall results were confirmed with a parallel analysis using hierarchical factor scores, in which the IUI-A and the IUI-B general factors reproduced quite well the expected divergent response patterns between clinical proxy groups and the reference group (Holaway et al., 2006; Gentes and Ruscio, 2011; Sternheim et al., 2011).

The current results support important avenues for future research regarding the interrelationships between IU, anxiety, depression, and comorbidity. The pattern suggests that targeting IU as a general risk factor may be beneficial at a global level, but when engaging treatment for a specific disorder (e.g., GAD, OCD) there may be benefits from targeting specific manifestations of IU. The contemporary transdiagnostic treatment models (e.g., the Unified Protocol; Ellard et al., 2010) may therefore be particularly well-suited as initial interventions, followed thereafter as necessary by disorder-specific modules (Grayson, 2010). The reverse order for treatment, starting with disorder-specific modules and then engaging transdiagnostic modules, may also be appropriate. In either case, the areas warrant additional research.

The current results also offer preliminary proof-of-concept evidence that using hierarchical factor scores to disentangle general and unique variance components could be useful to highlight common and specific characteristics of clinical-proxy samples (Reise, 2012). Nevertheless, the presence of a general IU factor represents a challenge for future research. On the one, hand the general factor might genuinely reflect IU-specific mechanisms that might account for diverse clinical manifestations of IU in a transdiagnostic framework. On the other hand, the general factor could merely represent a common method factor or some response set biases. Whatever the source of the common variance, group differences in the clinical manifestations of IU were overestimated when using summated ratings to assess non-clinical participants.

Different groups screening positive for anxiety and/or depression were not actually statistically different in some of the factor scores, as it was observed for “avoidance” and “need for control,” after controlling for the effect of the general IU variance. The result suggests that experiential avoidance and attempts to control uncertainty in anxious and depressed patients might be due to generalized IU core beliefs. If confirmed with clinical patients, these findings might suggest that IU core beliefs should be prioritized when treating patients reporting these specific clinical consequences of IU. By contrast, factor scores like “doubting” and “overestimation of threat” were still significant after controlling for the effect of the general IU variance. Not only the three clinical proxy groups were significantly higher than the reference group on “doubting” scores, but a follow up analysis revealed that the co-occurrence group was significantly higher than the depression and anxiety groups combined. The current results, if confirmed with clinical patients, might suggest that both IU core beliefs and doubting should be prioritized when treating patients reporting this specific clinical consequence of IU.

The current study has limitations that also provide important directions for future research. First, the current study used established clinical tools and applied internationally valid cut-offs to identify participants reporting clinically significant symptoms. Nevertheless, a major constraint is the lack of clinical interviews, which would have provided more accurate information concerning the clinical status of the research participants. Therefore, it is no warranted that the findings of the study could be generalized to clinical patients. Indeed, future investigations should attempt to replicate the current results with data gathered from formally diagnosed participants, or adding clinical interviews to the research design. If replicated, the results would support more nuanced clinical utility for total, subscale, and factor scores. Second, despite robust psychometrics, the application of IUI-B subscale scores was undermined by the relatively low unique variance. The current results support deriving a total score through simple aggregation of items for each of the six subscales mostly to reflect general factor variance. Accordingly, use of the subscale scores as reliable indicators of specific constructs currently warrants caution (Chen et al., 2012). The factor scores from the bifactor model may be more reliable (Reise et al., 2010; Chen et al., 2012), but present challenges for practicality. Future researchers should consider developing applications to facilitate the practical utility of clinical factor scores for identifying general and specific (i.e., IUI-B) sources of variance in IU. Third, the incremental value of the IUI-B hierarchical factor scores over standard assessments of needs to be addressed in rigorous empirical investigations before the clinical implementation of this scoring method. Future researchers should consider developing larger and more diverse assessments of general and specific clinical manifestations to strengthen the incremental utility of specific IU sources (e.g., Thibodeau et al., 2015).

Notwithstanding the limitations, the current study contributes to cross-validation of the IUI beyond use with French and English Canadian samples and beyond North America. Our study provided psychometric support for the Italian version of the IUI scales and preliminary normative data for international clinical research on IU. Previous cross-validation efforts worldwide supported contemporary refinements for defining (see Carleton, 2016b) and assessing IU (e.g., use of the IUS-12; Helsen et al., 2013). Similarly, the current results suggest (1) an abridged ten-item version of the IUI-A as a promising candidate for transdiagnostic measurement of IU core beliefs in large assessment batteries; and (2) using factor scores may be appreciably more defensible than simple aggregates for measuring general and specific IU for clinical and experimental methods.

## Ethics Statement

All procedures performed in studies involving human participants were in accordance with the ethical standards of the institutional and/or national research committee and with the 1964 Helsinki declaration and its later amendments or comparable ethical standards. Informed consent was obtained from all individual participants included in the study

## Author Contributions

The authors discussed the contents of this article together. ML, OM, RF, CT and RT conceived the study. ML, OM and RNC elaborated on the theoretical framework and the research hypotheses. CT and RF collected the data. ML and RNC analyzed the data. The final version of the manuscript was written by ML, OM, CT and RNC.

## Conflict of Interest Statement

The authors declare that the research was conducted in the absence of any commercial or financial relationships that could be construed as a potential conflict of interest.
