# Supplementary material for: The Intolerance of Uncertainty Inventory: Validity and Comparison of Scoring Methods to Assess Individuals Screening Positive for Anxiety and Depression
Source: Front Psychol. 2018 Mar 26;9:388. doi: 10.3389/fpsyg.2018.00388 (PMC5879456; doi:10.3389/fpsyg.2018.00388)
Supplement: Supplementary file 1 [file Table_1.DOCX]

Supplementary Table 1.

The Italian translation of the Intolerance of Uncertainty Inventory items

| IUI-A 1* | Ho difficoltà ad accettare che il futuro sia incerto |
| --- | --- |
| IUI-A 2* | Trovo insopportabile non avere garanzie nella vita |
| IUI-A 3* | Gli altri sembrano tollerare l'incertezza meglio di me |
| IUI-A 4 | Trovo intollerabile che alcuni aspetti della vita non siano determinati in anticipo |
| IUI-A 5 | Ho difficoltà a tollerare la possibilità che mi possa accadere un evento negativo |
| IUI-A 6* | Quando aspetto una notizia importante trovo difficile rimanere all'oscuro |
| IUI-A 7 | Trovo intollerabile avere a che fare con situazioni non prevedibili |
| IUI-A 8 | Non riesco davvero a sopportare situazioni in cui non so cosa sta per succedere |
| IUI-A 9 | Non sapere in anticipo cosa accadrà è spesso inaccettabile per me |
| IUI-A 10 | I periodi di attesa mi sono insopportabili quando non so cosa sta per accadere |
| IUI-A 11 | Ho difficoltà a tollerare le incertezze nella vita |
| IUI-A 12 | Quando penso che qualcosa di negativo potrebbe accadere ho difficoltà a rimanere nell'incertezza |
| IUI-A 13 | Vorrei sapere tutto subito piuttosto che rimanere nell'incertezza |
| IUI-A 14 | Ho difficoltà ad affrontare la possibilità che qualcosa di inatteso possa accadere |
| IUI-A 15* | Devo essere sicuro di quello che porto avanti |
|  |  |
| IUI-B 1 | Preferisco evitare le situazioni incerte |
| IUI-B 2 | Quando mi trovo in una situazione incerta, tendo ad avere dubbi su quello che sto facendo |
| IUI-B 3 | Spesso esagero le probabilità che il peggio possa accadere quando succede qualcosa di imprevisto |
| IUI-B 4 | Tendo a voler comandare gli altri in modo che niente di imprevisto possa accadere loro |
| IUI-B 5 | Spesso mi affido alla rassicurazione degli altri quando non conosco cosa accadrà |
| IUI-B 6 | Mi preoccupo molto sulle incertezze della vita |
| IUI-B 7 | Spesso ho dubbi su me stesso quando una situazione è incerta |
| IUI-B 8 | La possibilità che un evento negativo possa accadere mi porta ad evitare alcune attività |
| IUI-B 9 | Quando sono incerto ho bisogno di essere rassicurato dagli altri |
| IUI-B 10 | Devo controllare ogni cosa per prevenire l'accadere di conseguenze negative |
| IUI-B 11 | Tendo a chiedere l'opinione altrui quando sono insicuro di quello che accadrà |
| IUI-B 12 | Evito situazioni in cui è probabile che possa accadere qualcosa di imprevisto |
| IUI-B 13 | Quando il risultato di un evento è incerto, spesso dubito di aver fatto tutto il necessario |
| IUI-B 14 | Quando un evento negativo potrebbe accadere spesso sovrastimo la probabilità che possa verificarsi |
| IUI-B 15 | Tendo a preoccuparmi quando sono incerto di quello che accadrà |
| IUI-B 16 | Chiedo spesso la stessa informazione a diverse persone per rassicurarmi su ciò che accadrà |
| IUI-B 17 | Le situazioni incerte mi preoccupano |
| IUI-B 18 | Quando sono incerto su ciò che accadrà cerco di controllare ogni cosa |
| IUI-B 19 | Tendo a sovrastimare la probabilità che qualcosa di male possa verificarsi quando non so cosa succederà |
| IUI-B 20 | Pensare che qualcosa di inatteso possa accadere mi preoccupa |
| IUI-B 21 | Quando sono incerto tendo a dubitare delle mie capacità |
| IUI-B 22 | Tendo a non impegnarmi in attività che comportano qualche incertezza |
| IUI-B 23 | Quando sono incerto tendo a sovrastimare la probabilità che gli eventi finiscano male |
| IUI-B 24 | Tendo a controllare le attività di una persona cui voglio bene per far diminuire le possibilità che possa accadergli qualcosa |
| IUI-B 25 | Anche se è inverosimile che un evento negativo possa accadere ho bisogno che mi venga detto ripetutamente che tutto andrà bene |
| IUI-B 26 | Preferisco abbandonare un progetto piuttosto che convivere con la sua incertezza |
| IUI-B 27 | Preferisco controllare tutto per diminuire l'incertezza |
| IUI-B 28 | Mi preoccupa non sapere cosa mi riserva il futuro |
| IUI-B 29 | In una situazione incerta tendo ad esagerare le probabilità che le cose possano andare male |
| IUI-B 30 | Spesso tendo a mettere in discussione le mie scelte quando sono incerto su quello che accadrà |

Notes: * indicates an item excluded from the final IUI-A analyses. Response scale: 1 (‘Non mi descrive per niente’) to 5 (‘Mi descrive perfettamente’).
