# Supplementary material for: The Intolerance of Uncertainty Inventory: Validity and Comparison of Scoring Methods to Assess Individuals Screening Positive for Anxiety and Depression
Source: Front Psychol. 2018 Mar 26;9:388. doi: 10.3389/fpsyg.2018.00388 (PMC5879456; doi:10.3389/fpsyg.2018.00388)
Supplement: Supplementary file 2 [file Table_2.docx]

Supplementary Table 2

Descriptive statistics and scoring key for the IUI subscale and total scores.

| Score | Total Sample (N=1046) | | Males (N=414) | | Females (N=627) | |
| --- | --- | --- | --- | --- | --- | --- |
|  | *M* | *SD* | *M* | *SD* | *M* | *SD* |
| *IUI-B* |  |  |  |  |  |  |
| Avoidance | 13.09 | 4.20 | 12.79 | 3.87 | 13.27 | 4.40 |
| Doubting | 14.05 | 4.60 | 12.88 | 4.39 | 14.80 | 4.59 |
| Overestimation | 13.43 | 5.33 | 12.84 | 5.22 | 13.84 | 5.37 |
| Worrying | 14.67 | 4.92 | 13.73 | 4.81 | 15.27 | 4.91 |
| Control | 14.23 | 5.13 | 14.62 | 5.03 | 13.96 | 5.19 |
| Reassurance | 13.74 | 4.61 | 12.68 | 4.26 | 14.42 | 4.71 |
| Negative Manifestations of Uncertainty | 82.97 | 23.99 | 79.31 | 3.75 | 85.30 | 24.64 |
| *IUI-A* |  |  |  |  |  |  |
| General Unacceptability of Uncertainty (10 items) | 27.69 | 9.31 | 26.11 | 22.61 | 28.69 | 9.21 |
| General Unacceptability of Uncertainty (15 items) | 43.23 | 12.74 | 40.93 | 12.65 | 44.68 | 12.60 |
|  |  |  |  |  |  |  |

Avoidance = IUI-B 12 + IUI-B 22 + IUI-B 26 + IUI-B 1+ IUI-B 8;

Doubting = IUI-B 21 + IUI-B 7 + IUI-B 13 + IUI-B 2 + IUI-B 30;

Overestimation = IUI-B 29 + IUI-B 14 + IUI-B 19 + IUI-B 3 + IUI-B 23;

Worrying = IUI-B 28 + IUI-B 6 + IUI-B 17 + IUI-B 15 + IUI-B 20;

Control = IUI-B 27 + IUI-B 10 + IUI-B 18 + IUI-B 4 + IUI-B 24;

Reassurance = IUI-B 9 + IUI-B 5 + IUI-B 11 + IUI-B 25 + IUI-B 16;

Negative Manifestations of Uncertainty = Avoidance + Doubting + Overestimation + Worrying + Control + Reassurance;

General Unacceptability of Uncertainty (10 items) = IUI-A 4 + IUI-A 5 + IUI-A 7 + IUI-A 8 + IUI-A 9 + IUI-A 10 + IUI-A 11 + IUI-A 12 + IUI-A 13 + IUI-A 14;

General Unacceptability of Uncertainty (15 items) = IUI-A 1 + IUI-A 2 + IUI-A 3 +IUI-A 4 + IUI-A 5 + IUI-A 6 + IUI-A 7 + IUI-A 8 + IUI-A 9 + IUI-A 10 + IUI-A 11 + IUI-A 12 + IUI-A 13 + IUI-A 14 + IUI-A 15.
